# Comorbidity of Anxiety and Depression With Hypertension Among Young Adults in the United States: A Systematic Review of Bidirectional Associations and Implications for Blood Pressure Control

**DOI:** 10.7759/cureus.88532

**Published:** 2025-07-22

**Authors:** Benjamin O Akangbe, Folasade E Akinwumi, Damilola O Adekunle, Aliyu A Tijani, Oluchi B Aneke, Stella Anukam

**Affiliations:** 1 Public Health, Georgia State University, Atlanta, USA; 2 Nutrition, University of Lincoln, Lincoln, GBR; 3 Social and Behavioral Health Sciences, Baylor University, Waco, USA; 4 Education, Texas Juvenile Justice Department, Austin, USA; 5 Biomedical Sciences, Georgia State University, Atlanta, USA; 6 General Practice, National Defence College Hospital, Abuja, NGA

**Keywords:** anxiety, chronic disease, hypertension, mental health, public health

## Abstract

Hypertension is a significant risk factor for cardiovascular diseases, while anxiety and depression are highly prevalent mental health disorders that may influence the development and management of hypertension. The bidirectional associations between these conditions remain understudied, particularly among adults in the United States. Understanding the interplay of mental health and hypertension is critical for improving clinical and public health interventions.

This systematic review aims to examine the prevalence and bidirectional associations between anxiety, depression, and hypertension among US adults; identify clinical, behavioral, and sociodemographic factors influencing comorbidity; and explore implications for hypertension management. Following the Preferred Reporting Items for Systematic Reviews and Meta-Analyses (PRISMA) guidelines, a comprehensive search was conducted across multiple databases, including PsycINFO, Scopus, PubMed, Web of Science, ScienceDirect, and Google Scholar, covering literature from 2015 to 2024. Eligible studies included cross-sectional, cohort, and case-control designs focusing on US adults (≥18 years) and examining the association between anxiety, depression, and hypertension. Data extraction covered study characteristics, diagnostic criteria, statistical findings, and relevant confounders. The Newcastle-Ottawa Scale (NOS) was used for quality assessment. Eight studies met the inclusion criteria, comprising six cross-sectional and two cohort studies. Anxiety and depression were significantly associated with increased hypertension risk, with stronger effects observed among low-income populations, women, and minority groups. Cohort studies indicated that depression contributed to hypertension incidence via inflammatory and autonomic dysfunction pathways, while cross-sectional studies highlighted that hypertension itself exacerbated psychological distress, leading to a cyclical comorbid relationship. The review also found that individuals with comorbid anxiety or depression had poorer hypertension control and lower adherence to antihypertensive treatment. The findings underscore the need for integrated care approaches that address both mental health and hypertension, particularly in vulnerable populations. Routine mental health screenings should be incorporated into hypertension management strategies to improve adherence and outcomes. Future longitudinal research should explore causal mechanisms and assess intervention effectiveness in mitigating the adverse effects of comorbidity.

## Introduction and background

Hypertension is a major modifiable risk factor of cardiovascular disease and a premature mortality factor with a rapidly increasing adult epidemiology in the United States [1]. Anxiety and depression are also rising in similar proportions as the most common mental health disorders, contributing significant clinical and public health burdens [2]. The coexistence of these conditions complicates the diagnosis, treatment, and long-term management of affected individuals, thus worsening public health outcomes [3].

This comorbidity is a major challenge in both clinical and public health perspectives. Research has shown that depression and anxiety decrease medication adherence in hypertension patients and thus promote maladaptive health habits such as maintaining substance abuse, poor diet, and physical inactivity [4,5]. Conversely, uncontrolled hypertension also places a chronic stress and physical burden that intensify psychological distress, leading to a cycle of self-perpetuating ill-health [6]. Therefore, addressing this relationship between behavioral, biological, and healthcare system factors requires a thorough understanding of contributory system factors [7].

Adulthood is a critical period for intervention that is often characterized by asymptomatic hypertension, causing a delay in diagnosis and treatment. In the same vein, mental disorders may be dismissed or undertreated due to the associated stigma or lack of access to mental health services [8]. Additionally, adults experience economic hardships such as financial instability, employment stress, and complex healthcare navigation that may increase the risk of hypertension and mental health disorders [9]. Despite the abundance of academic literature focused on mental disorders and chronic illnesses, little is understood about the bidirectional relationship in adults, a population that may require distinct prevention and treatment strategies. This gap in knowledge underscores the need for a synthesized evidence reporting the relationships between anxiety, depression, and hypertension among this age group and effective clinical and public health measures that may ameliorate these outcomes.

This systematic review aims to assess the distribution and bidirectional associations between anxiety and depression in hypertension among adults in the United States; identify clinical, behavioral, and healthcare factors associated with comorbidity; and explore implications for hypertension management, including developing strategies to enhance mental healthcare in routine clinical practice for adults. This review synthesizes current evidence to inform targeted, multidisciplinary interventions to improve health outcomes for this population and guide public health strategies for this age group.

## Review

Methods

Study Design

This systematic review follows the guidelines of Preferred Reporting Items for Systematic Reviews and Meta-Analyses (PRISMA) [10] to ensure transparency and reproducibility. It reviews relevant literature on the comorbidity of anxiety, depression, and hypertension in US adults to examine prevalence, bidirectional associations, associated factors, and management strategies.

Data Sources and Search Strategy

The search employed a comprehensive, multi-database strategy. PubMed, Scopus, Web of Science, Excerpta Medica dataBASE (EMBASE), Cumulative Index to Nursing and Allied Health Literature (CINAHL), American Psychological Association (APA) PsycArticles, Cochrane Library, ScienceDirect, Google Scholar, and PsycINFO were searched with a comprehensive search for studies from January 2015 to March 2024. A combination of Medical Subject Headings (MeSH) and free text terms for “anxiety”, “depression”, “hypertension”, “comorbidity”, “mental health”, and “adults”, connected by Boolean operators (AND/OR), was used in the search strategy. Besides database searches, the reference lists of included studies were screened manually for capturing any additional relevant articles. Sources excluded were non-peer-reviewed, such as grey literature, dissertations, and conference abstracts, to ensure data reliability and consistency.

Study Selection

Titles and abstracts were independently screened by two reviewers, with discrepancies resolved through discussion or consultation with a third reviewer. Full-text assessments were then conducted to determine final eligibility. A total of 358 records were initially retrieved from electronic databases. After removing 132 duplicate or ineligible entries, 226 articles remained for title and abstract screening. Of these, 38 full-text articles were sought for further evaluation; however, 27 were excluded due to outdated publication dates or lack of relevant data. Ultimately, eight studies met the predefined inclusion criteria and were selected for final analysis, comprising six cross-sectional and two cohort studies. No case studies were included, as none met the eligibility requirements for this systematic review.

Inclusion and Exclusion Criteria for Study Selection

The inclusion and exclusion criteria applied during the study selection process are detailed in Table 1. Studies were considered eligible for inclusion if they involved adult participants aged 18 years or older diagnosed with hypertension; reported on the prevalence or comorbidity of anxiety in hypertensive patients; employed observational designs such as cross-sectional, cohort, or case-control studies; were published in English; provided full-text peer-reviewed articles; and used standardized diagnostic tools such as the Diagnostic and Statistical Manual of Mental Disorders, Fifth Edition (DSM-5), or the International Classification of Diseases, Tenth Revision (ICD-10). In contrast, studies were excluded if they did not focus on hypertension or anxiety as primary outcomes; were case reports, letters to the editor, reviews, or editorials; involved non-human subjects; were not published in English; lacked full-text availability; or had incomplete or missing data on anxiety prevalence.

**Table 1:** Inclusion and Exclusion Criteria for Study Selection DSM-5: Diagnostic and Statistical Manual of Mental Disorders, ICD-10: International Classification of Diseases

Inclusion Criteria	Exclusion Criteria
Studies involving adult participants (≥18 years) diagnosed with hypertension	Studies not involving hypertension or anxiety as primary outcomes
Studies reporting on the prevalence or comorbidity of anxiety in hypertensive patients	Case reports, letters to the editor, reviews, and editorials
Observational studies (cross-sectional, cohort, or case-control designs)	Non-human studies (animal research)
Studies published in English	Studies not published in English
Peer-reviewed full-text articles available	Abstracts without full text
Articles that used standardized diagnostic tools, e.g., DSM-5 and ICD-10	Studies with incomplete or missing data on anxiety prevalence

Data Extraction

Data extraction was performed independently by two reviewers using a structured data collection form to ensure accuracy and consistency. All the extracted data covered a number of fundamental domains to make a thorough synthesis and analysis. The study characteristics examined include author(s), year of publication, country of origin, and the type of study design utilized. Demographic information, such as sample size, age ranges, gender distribution, and socioeconomic status, was outlined. Exposure and outcome definitions were made based on the established definition of diagnosed anxiety, depression, or hypertension, namely, the Diagnostic and Statistical Manual of Mental Disorders, Fifth Edition (DSM-5), the International Classification of Diseases, Tenth Revision (ICD-10), or using clinical measurements. Additional statistical parameters were also extracted, including prevalence rates, odds ratios, risk ratios, and confidence intervals that are relevant. Possible confounding factors were considered, and data were collected as to any changes made for age, sex, socioeconomic background, comorbidities, and other factors that influence the outcome. Results were summarized, and key findings were noted with regard to the nature and direction of reported associations, as well as limitations in the original study. Disagreements in data extraction were discussed and resolved by a third reviewer to ensure consensus.

Quality Assessment

The Newcastle-Ottawa Scale (NOS) for observational studies (cross-sectional, cohort, and case-control) was employed to evaluate risk of bias in all included studies. The NOS evaluates studies on three domains: selection of participants, comparability of groups, and assessment of outcome or exposure. Low, moderate, and high risk of bias were used to categorize the studies. Those considered to be high risk were excluded from the final synthesis.

Data Synthesis

The data synthesis approach combined both quantitative and qualitative findings in this systematic review. The thematic content of these studies was grouped into prevalence rates, bidirectional associations, and contributing factors. The numerical outcomes were also summarized with descriptive statistics where feasible. The consistency and discrepancies were highlighted to make an informed synthesis of data reported in all of the included studies.

Results

Summary of the Literature Search Process

**Figure 1:**
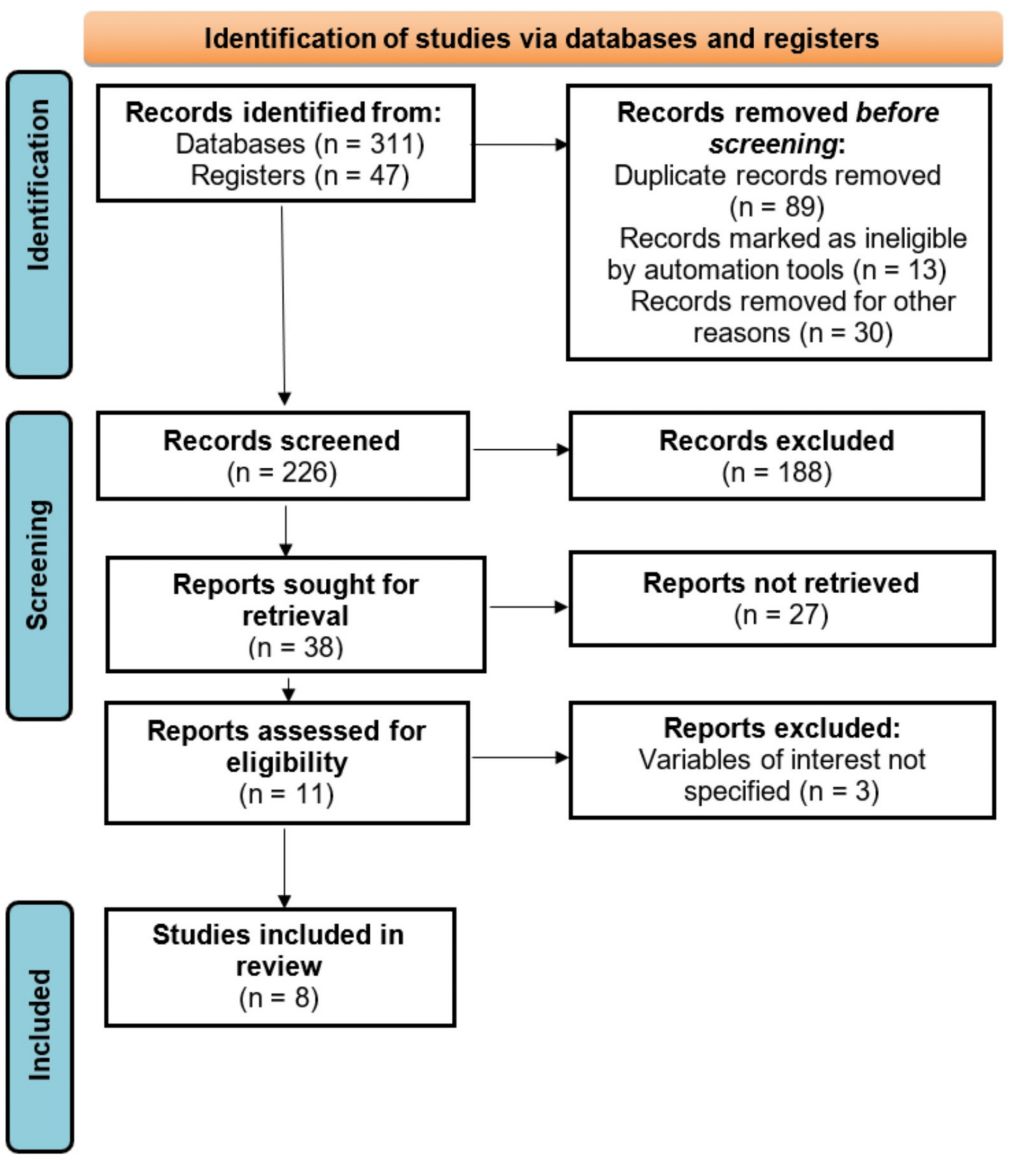
PRISMA Flow Diagram Summarizing Literature Search and Selection PRISMA: Preferred Reporting Items for Systematic Reviews and Meta-Analyses

The results of the systematic review indicated that a total of 358 records were initially identified through the comprehensive database search, as seen in Figure 1, which was obtained from the use of electronic academic databases such as PsycINFO, Scopus, PubMed, Web of Science, ScienceDirect, Latin American and Caribbean Health Sciences Literature (LILACS), ProQuest, EMBASE, Cochrane Library, Google Scholar, Health Management Information Consortium (HMIC), Cumulative Index to Nursing and Allied Health Literature (CINAHL), Medline, Open Grey, Campbell Collaboration, Allied and Complementary Medicine Database (AMED), and Database of Abstracts of Reviews of Effects (DARE). However, the focus was on PsycINFO, Scopus, PubMed, Web of Science, ScienceDirect, and Google Scholar to guarantee the collection of the most relevant and easily accessible academic literature for thorough review and extraction. After removing 132 records for reasons including duplication and ineligibility based on automated tools, 226 records were screened through title and abstract review. Of these, 188 were excluded for not meeting the inclusion criteria, and 38 full-text articles were retrieved for detailed evaluation. Subsequently, 27 studies were excluded due to being outdated or lacking relevant data, leaving 11 studies for full assessment. After further evaluation, three additional studies were excluded for not reporting on the variables of interest, resulting in eight final studies included in the review. This multistage screening process is detailed in Table 2.

**Table 2:** Summary of Literature Search and Selection Process

Stage	Number of Records
Records identified from databases	311
Records identified from registers	47
Total records identified	358
Duplicate records removed	89
Records removed by automation tools	13
Other records removed	30
Records screened	226
Records excluded after screening	67
Full-text articles assessed for eligibility	38
Full-text articles excluded	27
Studies excluded due to irrelevant variables	3
Studies included in the final systematic review	8

Table 2 provides a detailed breakdown of the literature selection process conducted in accordance with PRISMA guidelines. A total of 358 records were initially retrieved through database and registry searches. After removing duplicates and ineligible entries through automation tools and manual screening, 226 articles were deemed suitable for abstract-level review. Of these, 188 were excluded based on title and abstract relevance, and 38 were selected for full-text retrieval. However, 27 were excluded due to publication date or lack of relevant data, and three more were removed for not reporting key variables. Ultimately, eight studies met the eligibility criteria and were included in the final analysis. This multistep filtration process ensured the inclusion of high-quality and relevant studies for the systematic review.

Study Characteristics

Out of the eight included studies, six were cross-sectional [11-16] and two were cohort studies [17,18]. The design, populations, and methodology of the included studies were heterogeneous, and the review is a comprehensive overview of the comorbidity of anxiety, depression, and hypertension. The populations studied range from young adults to older adults, low-income populations, healthcare populations, and Native American groups. Study methodologies included cross-sectional studies, cohort studies, and/or longitudinal studies, capturing prevalence data in snapshot and longer-term associations. Various sample sizes were included for studies that varied in outcome measures (some measured self-reported data), and others measured clinical data [15]. This is also true for smaller, targeted groups [14], as well as studies with diverse gender distributions [12] and larger population-based studies [13]. Follow-up duration varied from cross-sectional snapshots [12] to multiple-year longitudinal studies [18].

The type of data collection method in the studies varied. Self-reported surveys were most frequently used to assess mental health symptoms, while hypertension data (blood pressure (BP)) was also sourced from medical record review. For example, as shown, some studies [14] focused on objective clinical measures, whereas others [16] used participant-reported data; the latter may be biased, but it provides better mapping in the medical care setting. Furthermore, longitudinal studies [18] included biological markers, for instance, inflammation data, which further improve understanding of the physiological pathways of the impact of mental health on hypertension.

Characteristics of the Included Studies

Table 3 summarizes the key characteristics of the eight studies included in this systematic review. The studies varied in design, with six adopting cross-sectional approaches and two employing cohort methodologies. The participant populations ranged widely, including young adults, older adults, low-income individuals, healthcare workers, and Native American groups, with age ranges spanning from 18 to over 65 years. Most studies had a relatively balanced gender distribution, with a slight female majority in all cases. The sample sizes also varied significantly, from smaller targeted groups of fewer than 200 participants to large-scale population-based studies exceeding 5,000 participants. Data collection methods included both clinical blood pressure (BP) readings and self-reported mental health assessments using validated tools such as the Generalized Anxiety Disorder 7-item (GAD-7) scale, the Patient Health Questionnaire-9 (PHQ-9), and the Center for Epidemiological Studies Depression (CES-D) scale. The follow-up durations differed across study types, with cross-sectional studies having no follow-up period and cohort studies extending up to eight years. Overall, Table 3 highlights the diversity in methodologies and population demographics across studies, reflecting the broad scope and generalizability of findings regarding the comorbidity among hypertension, anxiety, and depression.

**Table 3:** Characteristics of the Included Studies GAD-7: Generalized Anxiety Disorder 7-item, PHQ-9: Patient Health Questionnaire-9, BP: blood pressure, AHA: American Heart Association, CES-D: Center for Epidemiologic Studies Depression Scale, CRP: C-reactive protein

Study	Population	Age Range	Gender Breakdown	Study Design	Sample Size	Outcome Measure (Questionnaire/Scale)	Data Collection Method	Follow-Up Duration	Key Results
Niles and O’Donovan [13]	US adults	18-65+	54% female	Cross-sectional	5,103	Self-reported anxiety (GAD-7), depression (PHQ-9), hypertension (self-reported)	Self-reported surveys	None	Anxiety and depression were stronger predictors of hypertension than obesity or smoking.
Mok et al. [14]	Preclinical medical students	18-24	60% female	Cross-sectional	147	Clinical BP readings (systolic/diastolic, AHA guidelines)	Clinical BP examinations	None	Stage 2 hypertension prevalence doubled in students with anxiety compared to general young adults.
Gould et al. [12]	Older adults	65+	58% female	Cross-sectional	3,676	Self-reported multimorbidity (hypertension, anxiety, depression)	Self-reported surveys	None	Multimorbidity, including hypertension, was more prevalent among those with anxiety.
Crookes et al. [17]	Young adults	18-30	52% female	Cohort study	2,982	Self-reported depressive symptoms (CES-D scale), BP readings (AHA guidelines)	Self-reports and BP examinations	8 years	Depressive symptoms and antidepressant use increased hypertension risk.
Santoni et al. [18]	Native American adults	30-65	55% female	Cohort	1,542	Clinical BP (systolic/diastolic, AHA guidelines) and inflammation markers (CRP levels)	Clinical examinations and biomarker analysis	5 years	Depression predicted incident hypertension via inflammatory pathways.
Patterson et al. [15]	Young adults	18-30	59% female	Cross-sectional	4,931	Clinical BP readings (AHA guidelines), self-reported anxiety (GAD-7), depression (PHQ-9)	BP examinations and self-reports	None	Anxiety and depression are associated with poorer cardiovascular health, including hypertension.
Shah et al. [16]	Low-income adults	25-65	61% female	Cross-sectional	1,294	Self-reported mental health (GAD-7, PHQ-9) and hypertension (self-report)	Self-reports via surveys	None	Anxiety and depression increased hypertension prevalence, particularly in socioeconomically disadvantaged groups.
Ho et al. [11]	Adults in healthcare	20-65+	56% female	Cross sectional	1,927	Self-reported anxiety (GAD-7), depression (PHQ-9), BP control (medication adherence; medical records)	Self-reports and medical record reviews	None	Anxiety and depression are linked to poorer hypertension control due to reduced medication adherence.

Quality Assessment

The included studies were assessed using the Newcastle-Ottawa Scale (NOS) cross-sectional variant for cross-sectional studies and the cohort variant for use in cohort studies. The domains assessed by the NOS are the selection of study participants, comparability of study groups, and outcome assessment. The rating scale was 0-9 stars, in which the higher the value, the better the quality.

Quality Assessment of Cross-Sectional Studies (NOS)

Table 4 presents the quality assessment of the six cross-sectional studies included in this review, based on the Newcastle-Ottawa Scale (NOS) adapted for observational studies. The NOS evaluates each study across three core domains: selection of participants, comparability of study groups, and outcome assessment. Scores range from 0 to 9, with higher scores indicating better methodological quality. All six studies demonstrated moderate to high quality. Mok et al. [14] and Patterson et al. [15] received the highest scores (8 out of 9), indicating strong design, appropriate comparability, and valid outcome measures. Other studies, such as those by Ho et al. [11], Shah et al. [16], and Niles and O’Donovan [13], scored 7, reflecting good quality with minor limitations. Gould et al. [12] received a score of 6, suggesting moderate quality primarily due to lower comparability. Overall, the table indicates that most of the cross-sectional studies included were methodologically sound, lending credibility to their findings on the comorbidity of anxiety, depression, and hypertension.

**Table 4:** Quality Assessment of Cross-Sectional Studies (NOS) NOS: Newcastle-Ottawa Scale

Study	Selection (0-4)	Comparability (0-2)	Outcome (0-3)	Total Score (0-9)	Quality Interpretation
Niles and O’Donovan [13]	3	1	3	7	High quality
Mok et al. [14]	4	2	2	8	High quality
Gould et al. [12]	3	1	2	6	Moderate quality
Patterson et al. [15]	4	2	2	8	High quality
Shah et al. [16]	3	1	3	7	High quality
Ho et al. [11]	3	2	2	7	Moderate to high quality

Quality Assessment of Cohort Studies (NOS)

Table 5 evaluates the methodological quality of the two cohort studies included in this review using the Newcastle-Ottawa Scale (NOS) for cohort studies. This tool assesses studies across three domains: selection of participants, comparability of study groups, and outcome assessment, with a maximum score of 9. Both cohort studies, Crookes et al. [17] and Santoni et al. [18], achieved high-quality ratings, scoring 8 and 9, respectively. Crookes et al. [17] scored well across all domains, with minor limitations in outcome assessment. Santoni et al. [18] received a perfect score, reflecting robust participant selection, excellent control for confounding factors, and thorough outcome evaluation, including the use of biological markers. These high scores indicate strong methodological rigor and support the reliability of the longitudinal findings linking depression to the development of hypertension. In general, the studies were considered of moderate to high quality. Cross-sectional studies tended to show variability in outcome measurement due to reliance on self-reported data, while cohort studies displayed greater robustness in longitudinal follow-up and biomarker inclusion. Although there were some limitations, the results are reliable due to the consistent use of validated mental health scales and clinical blood pressure (BP) assessments.

**Table 5:** Quality Assessment of Cohort Studies (NOS) NOS: Newcastle-Ottawa Scale

Study	Selection (0-4)	Comparability (0-2)	Outcome (0-3)	Total Score (0-9)	Quality Interpretation
Crookes et al. [17]	4	2	2	8	High quality
Santoni et al. [18]	4	2	3	9	High quality

Prevalence of Comorbidity

The result of this systematic review showed that regardless of demographics, the comorbid prevalence of anxiety, depression, and hypertension was relatively high across all ages, socioeconomic statuses, and clinical settings, showing different patterns by age and clinical setting but similarity by socioeconomic status. When anxiety or depression coexisted, there was an early onset of hypertension in young adults [14,15], implying that psychological distress may accelerate cardiovascular risk even in populations thought for so long to be low risk. In older adults [12], the prevalence of multimorbidity has soared, coupled with elevated anxiety and depressive symptoms on top of hypertension, further worsening global health. Comorbidity rates varied among low-income populations [16], which may be indicative of systemic healthcare barriers, additional life stressors, and environmental factors, including food insecurity and lack of access to preventive care. Santoni et al. [18] have also noted the higher prevalence of hypertension in Native American adults with depression, which further supports the need to explore racial and ethnic disparities. The emphasis on these findings is that comorbidity is not evenly distributed and should be accounted for in early detection and targeted interventions.

Table 6 provides an overview of the prevalence findings from the selected studies concerning the co-occurrence of anxiety, depression, and hypertension. The table shows consistent evidence across populations that comorbidity is notably prevalent and clinically significant. For example, Niles and O’Donovan [13] found that anxiety and depression were stronger predictors of hypertension than even well-known risk factors such as obesity and smoking. Similarly, Mok et al. [14] reported that stage 2 hypertension was twice as common among preclinical medical students who experienced anxiety compared to the general young adult population. Patterson et al. [15] also noted a strong association between poor cardiovascular health and elevated anxiety and depressive symptoms in young adults. These findings suggest that psychological distress can contribute significantly to hypertension risk and that mental health conditions may serve as early indicators of cardiovascular vulnerability, especially among younger and socioeconomically disadvantaged groups.

**Table 6:** Prevalence of Comorbidity

Study	Population	Age Range	Prevalence of Comorbidity Findings
Niles and O’Donovan [13]	US adults	18-65+	Anxiety and depression are more predictive of hypertension than obesity or smoking.
Mok et al. [14]	Preclinical students	18-24	Stage 2 hypertension prevalence doubled among anxious medical students.
Patterson et al. [15]	Young adults	18-30	Poor cardiovascular health, including hypertension, is linked to higher anxiety and depressive symptoms.

Bidirectional Associations

Several studies showed that the relationship between anxiety, depression, and hypertension is bidirectional. Crookes et al. [17] and Niles and O’Donovan [13] have shown that anxiety and depression lead to sustained sympathetic nervous system activation and contribute to the development of hypertension. Conversely, Shah et al. [16] and Patterson et al. [15] demonstrated that hypertension itself is a chronic stressor that heightens mental health conditions in vulnerable, low-income populations. The cyclical relationship suggests that effective treatment should be a balanced combination of methods that simultaneously address mental health and cardiovascular outcomes.

Table 7 illustrates the bidirectional relationship observed between anxiety, depression, and hypertension across several studies. The findings demonstrate that psychological disorders not only contribute to the onset of hypertension but that hypertension itself may intensify mental health challenges, particularly in vulnerable populations. Studies such as those by Crookes et al. [17] and Niles and O’Donovan [13] provide evidence that anxiety and depressive symptoms, along with antidepressant use, can increase the risk of developing hypertension through mechanisms such as sympathetic nervous system activation and autonomic dysfunction. Conversely, Shah et al. [16] and Patterson et al. [15] highlight that living with hypertension, particularly under chronic stress and low socioeconomic conditions, can exacerbate anxiety and depression. This cyclical, self-reinforcing pattern underscores the importance of integrated healthcare strategies that simultaneously address both mental and cardiovascular health to break the comorbidity cycle and improve overall outcomes.

**Table 7:** Bidirectional Associations

Study	Anxiety/Depression Leading to Hypertension	Hypertension Leading to Mental Health Issues
Niles and O’Donovan [13]	Sustained stress and sympathetic nervous system activation.	-
Crookes et al. [17]	Depressive symptoms and antidepressant use are linked to increased hypertension.	-
Shah et al. [16]	-	Hypertension is linked to greater anxiety and depression rates in low-income adults.
Patterson et al. [15]	-	Poor cardiovascular health worsens mental health outcomes.

Subgroup and Sociodemographic Factors

The subgroup analyses showed that the comorbidity rate was dependent on gender, socioeconomic status, race, and healthcare access. The incidence of anxiety-driven hypertension was consistently greater in women than in men [12], possibly due to gender disparities in emotional regulation and hormonal processing, as well as in healthcare-seeking behavior. Particularly, socioeconomic disparities were revealed [16], where lower-income adults have more barriers to psychosocial services, delayed diagnosis of hypertension, and lower medication adherence. In addition, while nearly all individuals accessed healthcare, there were healthcare access limitations that were prominent: uninsured or underinsured respondents experienced higher rates of comorbidity, related to less access to prevention care. For example, the highest rates of depression linked to hypertension were found in Native American adults [18], which was due to structural inequities and cultural barriers to healthcare. These subgroup findings highlight the importance of considering the individual barriers faced by marginalized and vulnerable populations to address specific barriers to successful management.

Table 8 highlights the influence of sociodemographic characteristics such as income level, gender, and ethnicity on the comorbidity of anxiety, depression, and hypertension. The included studies reveal clear disparities in prevalence and impact across different population subgroups. For instance, Shah et al. [16] found that low-income individuals were significantly more likely to experience all three conditions concurrently, reflecting the compounding effects of financial stress, reduced healthcare access, and environmental hardship. Gould et al. [12] observed that women exhibited higher rates of anxiety and multimorbidity, including hypertension, than men, likely due to both biological susceptibility and social role stressors. Furthermore, Santoni et al. [18] reported a disproportionately higher incidence of hypertension among Native American adults with depression, underscoring the role of racial and ethnic disparities rooted in structural inequities. These subgroup findings suggest that effective interventions must be tailored to the unique challenges faced by marginalized populations to reduce health inequities and improve care outcomes.

**Table 8:** Subgroup and Sociodemographic Factors

Study	Key Subgroup Findings
Shah et al. [16]	Low-income individuals are more likely to experience anxiety, depression, and hypertension concurrently.
Gould et al. [12]	Women exhibited higher rates of anxiety and multimorbidity, including hypertension, compared to men.
Santoni et al. [18]	Native American adults with depression showed increased hypertension incidence, indicating racial disparities.

Implications for Management

High comorbidity prevalence and how it varies between groups are emphasized as urgently needed integrated care models. Routine mental health screening in primary care settings, especially for patients with hypertension, should now be practiced. Mental health symptoms and cardiovascular risk can be mitigated by behavioral interventions such as cognitive behavioral therapy (CBT), stress reduction programs, and physical activity promotion. For comorbid conditions to be adequately integrated, it is vital that care coordination is improved between mental health providers, generalist medical practitioners, and cardiologists. Also, in order to expand healthcare to those in low-income and underserved populations, community-based initiatives are necessary to narrow the systemic inequalities in access to healthcare. Mental health awareness campaigns by public health must be seen as a priority, mental health services should be available in hypertension clinics, and social determinants of health, such as poverty and food insecurity, should be addressed to prevent the development and worsening of comorbidity. Going forward, digital health technologies such as telemedicine and mental health apps should be explored as potential adjuncts to long-term management and improve outcomes for patients faced with barriers to normal care.

Discussion

Hypertension is a major cause of morbidity and mortality globally, contributing to cardiovascular disease, stroke, and premature death [19]. Over the years, modifiable lifestyle factors such as obesity, smoking, and even physical inactivity have been described as the primary risk drivers of hypertension [12]. However, the role of mental health, particularly anxiety and depression, in hypertension development and management has received significant attention in recent years [20].

Anxiety and depression are prevalent among adults in the United States. According to the National Health Statistics Reports, approximately 6% of adults reported having moderate or severe symptoms of anxiety and 7% of adults reported having moderate or severe symptoms of depression in the past two weeks [21]. Despite this high prevalence, little consideration is often given to the effect these mental health conditions have on hypertension in clinical practice [22].

The significance of this study lies in its synthesis of existing evidence to elucidate the bidirectional associations between anxiety and depression and hypertension among US adults. Such relationships are important to understand in order to improve both mental healthcare and cardiovascular outcomes in, especially, relevant vulnerable groups, such as low-income groups and marginalized communities [23]. By highlighting the extent of comorbidity and the pathways linking mental health to hypertension, this review advocates for a shift toward more integrated, multidisciplinary approaches to healthcare [11].

This systematic review reveals a consistent and significant association between anxiety, depression, and hypertension among US adults. Across various populations and study designs, comorbidity rates were notably high, with mental health conditions emerging as strong predictors of hypertension. Cross-sectional studies, such as those by Patterson et al. [15] and Niles and O’Donovan [13], demonstrated that anxiety and depression were more predictive of hypertension than traditional risk factors such as obesity and smoking. Moreover, cohort studies, such as those by Crookes et al. [17] and Santoni et al. [18], reinforced this pattern by showing that depressive symptoms and inflammation pathways contributed to incident hypertension over time. Subgroup analyses further revealed that low-income adults, women, and Native American populations experienced higher rates of comorbidity, reflecting the intersection of mental health disparities and cardiovascular outcomes. Importantly, the quality assessment indicated that most studies were of moderate to high quality, strengthening the reliability of the observed associations despite limitations in self-reported data.

Despite extensive studies, it is unclear whether mental health disorders are associated with hypertension. The high prevalence rates of anxiety and depression among hypertensive individuals have been the focus of several studies; however, reported prevalence rates varied widely. For example, in another study, 26.8% of hypertensive patients were depressed, indicating a significant comorbidity [24]. Nonetheless, differences in study populations, diagnostic criteria, and methodological approaches result in significant differences in prevalence estimates [25]. Therefore, this indicates that demographic, cultural, and contextual factors influence the relationship between mental disorders and hypertension. This very notion is also supported by more recent research highlighting the influence of stress exposure, health behaviors, and socioeconomic factors on mental health outcomes in hypertensive patients [26].

The association of hypertension, anxiety, and depression remains a topic of contention in regard to directionality. According to some research, chronic stress and dysregulation of physiological processes may be triggered by mental health disorders, leading to hypertension development [22,27]. Higher sympathetic nervous system activity [28] has been associated with increased and sustained BP and vascular dysfunction, particularly as a result of chronic psychological distress (i.e., anxiety and depression). On the contrary, other studies posited that hypertension itself can induce or exacerbate anxiety and depression because of the psychological load of caring for a chronic illness [29,30]. The fact that causality exists in a bidirectional relationship establishes its inherent complexity and emphasizes the importance of longitudinal studies to analyze temporal associations and underlying mechanisms.

Several studies explored the biological mechanisms connecting mental health disorders and hypertension. The commonly proposed pathways are the dysregulation of the autonomic nervous system, hypothalamic-pituitary-adrenal (HPA) axis hyperactivity, and increased inflammatory responses [20]. Prolonged stress may result in increased vascular resistance, elevated BP, and endothelial dysfunction, which are associated with elevated cortisol levels [31]. Further, inflammatory markers such as C-reactive protein (CRP) are found to be elevated in both depression and hypertension, which suggests a common inflammatory factor between both conditions [31,32]. These findings thus add to the argument that mental health is crucial to hypertension risk and progression, and as such, targeted interventions for mental wellness should be combined with the management of traditional BP risk factors.

While disparities in hypertension prevalence and control are well established in cross-sectional studies across socioeconomic and ethnic groups, less attention has been paid to the mediating roles of anxiety and depression on these disparities. Studies have shown that lower income and minority populations are at higher risk of coexistent hypertension and mental health disorders [33]. The burden of depression in the African American community is associated with limited access to healthcare, financial stress, and systemic barriers [14]. However, other studies do not control for factors that can confound the relationship between hypertension and mental health outcomes, such as history of healthcare use and social network [34].

Race and ethnicity further complicate this relationship. Hypertension rates among African Americans are disproportionately higher than White adults, and these higher rates are related to socioeconomic and structural inequities and barriers to healthcare [13]. When coupled with increased anxiety and depression prevalence, particularly among individuals experiencing discrimination or economic hardship [35], the burden grows. Research indicates that chronic stress associated with racial discrimination is linked to sustained BP elevation and increases in cardiovascular risk [36]. Furthermore, African Americans may also have lower rates of mental health service utilization, which can exacerbate comorbidities and highlight the urgent need for culturally tailored interventions for the management of mental health and hypertension in high-risk communities [37].

Another complicating factor is that gender has been shown to relate differently to the link between hypertension and mental health. Studies show that hypertensive women are more subject to psychological distress than hypertensive men; however, women have a greater tendency for emotional problems of anxiety and depression as compared to men [15]. It is possible that the co-variation between hormonal fluctuations, social roles, and access to healthcare contributes to this disparity [38]. Furthermore, women with hypertension may also have heightened anxiety when it comes to disease management and treatment adherence, which is a risk meriting adverse cardiovascular outcomes [18]. Therefore, hypertension care models will benefit from sex-specific interventions in mental health, given this gender-based vulnerability [16].

Beyond biological and demographic influences, factors that are related to behavior also have an important role in the interrelationship between mental health disorders and hypertension. Anxiety and depression are associated with reduced adherence to antihypertensive medications and health-promoting behaviors such as regular exercise and balanced nutrition [39,40]. Individuals with mental health disorders have poor adherence to treatment regimens, which complicates hypertension management and worsens complications such as stroke and cardiovascular disease [41].

Healthcare access and provider engagement further influence comorbidity management. Fragmentation between mental health and primary care services often results in suboptimal treatment for individuals with both hypertension and mental health conditions [42]. Although there is evidence about integrated care models, many healthcare systems still do not integrate mental and physical healthcare, making holistic care for comorbidity more difficult [43]. Early detection and intervention for hypertension should be improved via the incorporation of mental health screening into routine care for hypertensive patients [44]. Furthermore, approaches that combine patient-centered approaches, such as shared decision-making and psychosocial support, may also facilitate adherence to mental health and hypertension treatment [45].

Altogether, this review highlights the interplay between anxiety, depression, and hypertension, which could be biological, behavioral, and social issue. Therefore, effective management of hypertension should reach beyond this limit and involve comprehensive mental health strategies that are specifically designed for vulnerable populations.

Implications for Policy and Practice

These findings have extreme relevance for policy and clinical practice in healthcare. Given the high prevalence of comorbid anxiety, depression, and hypertension, it is imperative that mental and physical healthcare delivery areas work together within the context of integrated care models. Current healthcare arrangements divide mental and cardiovascular health as two separate entities, as evidenced by fragmented care [11]. Mental health screening in routine hypertension management can help clinicians proactively detect the psychological strife that influences BP control [22].

Public health policies should prioritize access to mental health services, especially for low-income and minority populations, who are more affected by comorbidity [46]. Therefore, it necessitates expanding mental health services in primary care sites, as well as educating healthcare providers to be able to identify and treat anxiety and depression in the context of cardiovascular treatment. Research suggests that mental health professionals partnering with primary care physicians is an effective strategy in helping patients with chronic illnesses and coexisting mental health illnesses [11].

Similarly, community-based interventions should target the social determinants of comorbidity. The prevention of hypertension and mental illness is burdened by socioeconomic inequalities, racial discrimination, and reduced access to preventive healthcare [13].

Guidelines for hypertension management should be clinically updated to reflect the two directions of the relationship between mental health and cardiovascular disease. It should include recommendations on a generic assessment routine for hypertensive patients and provision of evidence-based interventions or psychotherapy (cognitive behavioral therapy (CBT) or antidepressants) to improve psychological state and BP control [39].

Strengths and Limitations of the Study

The most important strength of this review is in its comprehensive synthesis of a number of varied study designs, populations, and methodologies. In including both cross-sectional and cohort studies, the review presents a more multidimensional view of the comorbidity among anxiety, depression, and hypertension. Such an exploration also offers biological, behavioral, and sociodemographic pathways into the analysis that further strengthen it, offering a multidimensional understanding of the issue.

Nevertheless, this review also has some limitations. Generally, the variation in the study designs, population characteristics, and outcome measures limits direct comparison between studies. Some studies rely on self-reported data and are therefore susceptible to recall bias or social desirability effect [37]. Furthermore, the findings cannot be generalized to other cultures or healthcare settings other than US adults [47,48].

Although these limitations limit the power to draw conclusions, the observed associations are consistent across studies, establishing the robustness of the findings. Future research should explore longitudinal data to define the direction of the relationship and develop an intervention strategy as a function of the high-risk population. Finally, policymakers and healthcare practitioners alike must understand the interaction and, as is true for other chronic illnesses, the intertwining health of people experiencing mental and cardiovascular illnesses, and this must be a part of a more holistic and equitable approach to patient care.

## Conclusions

This systematic review highlights the complex, reciprocal relationship between anxiety, depression, and hypertension among US adults. The consistent evidence across diverse populations and study designs indicates that mental health disorders are not only common among individuals with hypertension but also act as significant, modifiable contributors to both its onset and poor management. Anxiety and depression influence hypertension through biological pathways such as inflammation and autonomic dysregulation, as well as behavioral mechanisms, including poor medication adherence and unhealthy lifestyle choices. Conversely, living with uncontrolled hypertension can exacerbate psychological distress, creating a self-reinforcing cycle of comorbidity. These findings challenge the traditional separation of physical and mental healthcare, emphasizing the need for more integrated and interdisciplinary approaches.

To improve outcomes, future healthcare models must prioritize the integration of mental health screening and treatment into routine hypertension management, particularly for vulnerable populations such as low-income groups, women, and racial minorities. Longitudinal research is essential to establish causal relationships and guide the development of targeted interventions that address both psychological and physiological dimensions of hypertension. Digital health technologies, such as telemedicine and mobile mental health tools, may offer promising avenues for expanding access to care in underserved communities. Ultimately, addressing comorbid anxiety and depression is vital not only for optimizing hypertension control but also for promoting overall well-being and health equity.
